# The Influence of Differentially Expressed Tissue-Type Plasminogen Activator in Experimental Autoimmune Encephalomyelitis: Implications for Multiple Sclerosis

**DOI:** 10.1371/journal.pone.0158653

**Published:** 2016-07-18

**Authors:** Lisa CM Dahl, Zeyad Nasa, JieYu Chung, Be’eri Niego, Volga Tarlac, Heidi Ho, Adam Galle, Steven Petratos, Jae Young Lee, Frank Alderuccio, Robert L. Medcalf

**Affiliations:** 1 Molecular Neurotrauma and Haemostasis, Australian Centre for Blood Diseases, Central Clinical School, Monash University, Melbourne, VIC, 3004, Australia; 2 Department of Immunology, Central Clinical School, Monash University, Melbourne, VIC, 3004, Australia; 3 Van Cleef Roet Centre for Nervous Diseases, Central Clinical School, Monash University, Melbourne, VIC, 3004, Australia; 4 Department of Medicine, Central Clinical School, Monash University, Melbourne, 3004, Victoria, Australia; Instituto de Investigaciones Biomedicas de Barcelona, CSIC, SPAIN

## Abstract

Tissue type plasminogen activator (t-PA) has been implicated in the development of multiple sclerosis (MS) and in rodent models of experimental autoimmune encephalomyelitis (EAE). We show that levels of t-PA mRNA and activity are increased ~4 fold in the spinal cords of wild-type mice that are mice subjected to EAE. This was also accompanied with a significant increase in the levels of pro-matrix metalloproteinase 9 (pro-MMP-9) and an influx of fibrinogen. We next compared EAE severity in wild-type mice, t-PA^-/-^ mice and T4+ transgenic mice that selectively over-express (~14-fold) mouse t-PA in neurons of the central nervous system. Our results confirm that t-PA deficient mice have an earlier onset and more severe form of EAE. T4+ mice, despite expressing higher levels of endogenous t-PA, manifested a similar rate of onset and neurological severity of EAE. Levels of proMMP-9, and extravasated fibrinogen in spinal cord extracts were increased in mice following EAE onset regardless of the absence or over-expression of t-PA wild-type. Interestingly, MMP-2 levels also increased in spinal cord extracts of T4+ mice following EAE, but not in the other genotypes. Hence, while the absence of t-PA confers a more deleterious form of EAE, neuronal over-expression of t-PA does not overtly protect against this condition with regards to symptom onset or severity of EAE.

## Introduction

Multiple sclerosis (MS) is an inflammatory and degenerative disorder of the central nervous system (CNS), characterized by infiltrating immune cells, demyelination and axonal damage. The aetiology is still unknown, although growing evidence has implicated changes in the blood brain barrier (BBB) permeability in the pathology of this disorder [1–3] that in turn facilitates immune cell migration into the CNS to propagate tissue damage. Various proteases, including the serine protease tissue-type plasminogen activator (t-PA) and matrix metalloproteinases (MMPs)-2 and 9 [4, 5] have been implicated in both BBB disruption and in the pathogenesis of MS. All MMPs are produced as zymogens or pro-forms that require proteolytic activation. Activation of a significant number of these enzymes is plasmin-dependent, indicating a functional interaction between the MMP and plasminogen activating enzyme systems. Therefore, it seems reasonable to hypothesise that increases in t-PA and subsequent plasmin activities would affect MMP levels and the progression of MS.

The MMPs appear to play a direct role in BBB disruption by acting upon basement membrane proteins within the neurovascular unit [6–9]. On the other hand, t-PA-mediated increase in BBB permeability can occur via both plasmin-dependent [10] and independent [11] processes. Moreover, t-PA-mediated plasmin formation can activate a number of MMPs including MMP-2, -3, -9, -12 and -13 [12–14], supporting a role for the plasmin-MMP axis in modulating neurovascular unit permeability.

t-PA has also been implicated in other aspects of brain function including promotion of memory formation [15], plasticity [16], cerebral glucose uptake [17], neurotoxicity [18] and neurodegeneration [19]. MS represents a particularly interesting condition in which t-PA appears to act in a protective capacity as extrapolated from animal models. Two previous studies evaluated t-PA^-/-^ mice subjected to MOG_35-55_-induced experimental autoimmune encephalomyelitis (EAE) [20, 21]. While both studies reported that t-PA^-/-^ mice had a more severe form of disease, there was a discrepancy in the time of disease onset. Nonetheless, consistent with a protective effect of t-PA in EAE, the incidence and clinical severity of EAE were both reduced in mice deficient in plasminogen activator inhibitor (PAI)-1, a naturally occurring t-PA inhibitor [22]. High levels of t-PA have been reported in the cerebrospinal fluid of MS patients [23, 24] suggesting heightened fibrinolysis in this condition, presumably as a protective measure. t-PA has also been shown to co-localize with fibrin on demyelinated axons in human MS [25] and deposited perivascularly in mouse models of encephalomyelitis [26].

Plasma depletion of fibrinogen with either ancrod or bathroxobin has been shown to be protective in mice subjected to EAE [27, 28]. As fibrin formation has been reported to inhibit remyelination [29], it has been suggested that t-PA may be engaging its classical fibrinolytic role to remove fibrin deposits from the CNS. Despite these observations, other studies have indicated that the fibrinolytic capacity of t-PA is negated in MS due to the increase in levels of PAI-1 with the concomitant increase in t-PA:PAI-1 complexes [30]. While complex formation is conventionally associated with protease clearance, t-PA:PAI-1 complexes themselves can initiate opening of the BBB in models of traumatic brain injury via activation of LDL receptor signalling [31]. However, whether t-PA:PAI-1 complex formation has any bearing on the pathogenesis of MS remains to be determined.

The observation that t-PA^-/-^ mice display more severe clinical EAE symptoms is also consistent with a role for t-PA in removing fibrin deposits on demyelinated axons in this model, but it argues against a deleterious role for t-PA in MS progression if this protease was acting primarily on the BBB. In this study, we evaluated changes in endogenous MMP and t-PA activity and expression in the spinal cord of mice subjected to EAE. Furthermore, we also compared the rate of onset and degree of EAE severity in t-PA^-/-^ mice as well as transgenic (T4+) mice that selectively over-express t-PA in the CNS [32]. Our findings confirm that t-PA deficiency results in a more accelerated rate of EAE onset, consistent with the earlier findings of East et al [21], and a more severe form of disease progression. In contrast, T4+ mice demonstrated a similar rate of onset and severity of EAE compared to wild-type control mice suggesting that neuronal overexpression of t-PA offers no advantage in the host response to EAE. ProMMP-9 levels also increased in spinal cord extracts to a similar degree in all genotypes following EAE. Curiously, MMP-2 levels were not increased in any of the mouse genotypes except in T4+ mice following EAE. Similarly, we also found that fibrinogen levels accumulated in the spinal cord of mice following EAE, and this also occurred to a similar extent in t-PA^-/-^ and in T4+ mice arguing against an overt effect of t-PA on BBB permeability during EAE. Hence, although endogenous t-PA is protective in EAE, overexpression of t-PA does not confer additional benefit to this condition.

## Materials and Methods

### Animals

All animal experiments were approved by the Alfred Medical Research and Education Precinct (AMREP) Animal Ethics Committee (approval #E/1355/2013M). Studies were performed using 8–15 week old male C57/Bl6-J mice. T4 mice are heterozygous transgenic mice that constitutively produce increased t-PA activity in post-natal neurons [33]. Mice were generated from crossing T4 heterozygous mice with wild-type C57/Bl6-J mice. From the litters, male T4 mice (T4+) and wild-type male littermate control mice (T4^control^) were selected by genotyping and used in this study. Male t-PA^-/-^ mice (backcrossed 13 generations against C57/Bl6-J mice) were obtained from a homozygote breeding line.

### Induction of experimental autoimmune encephalomyelitis (EAE)

**Fig 1:**
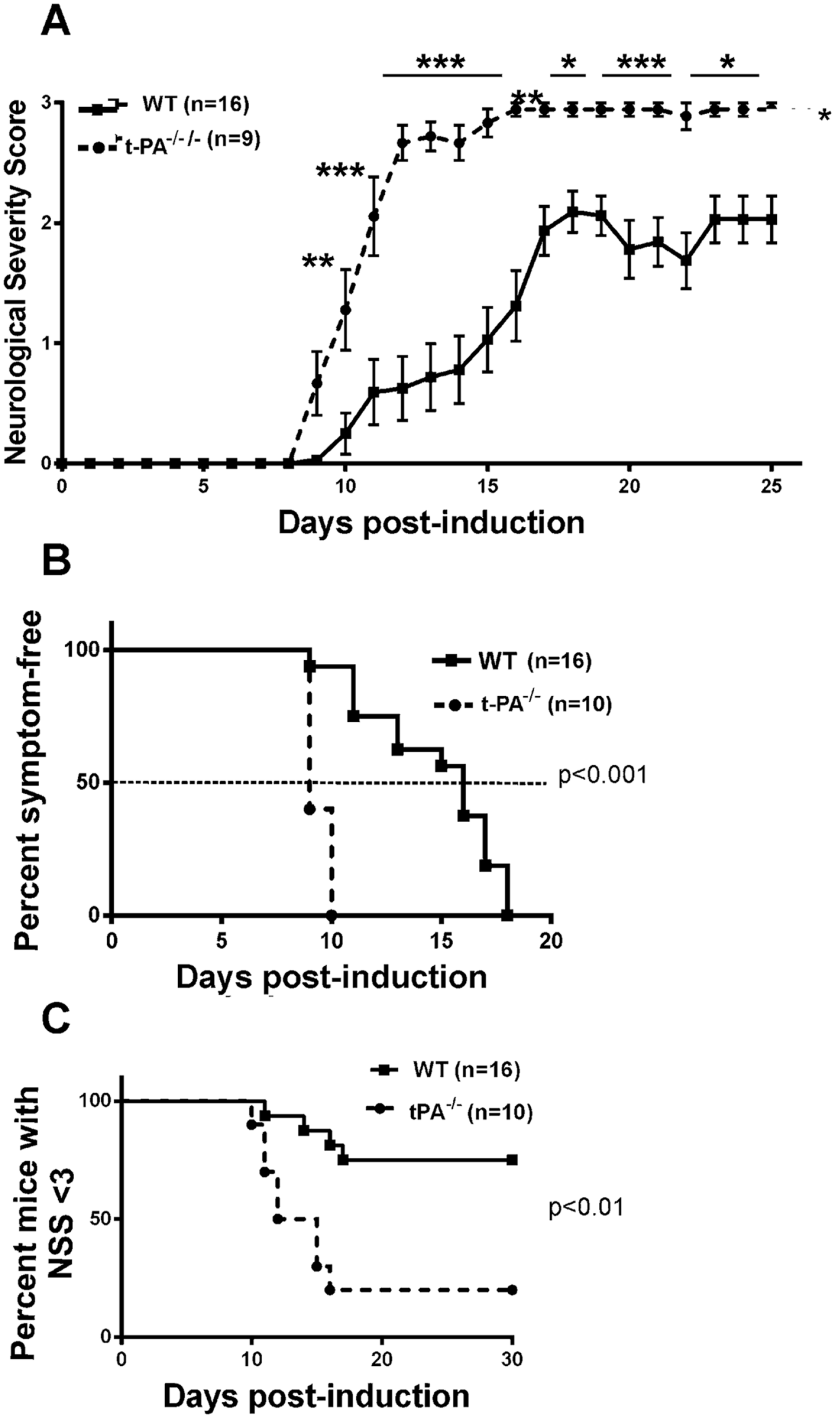
t-PA^-/-^ mice develop a more severe form of EAE. (A): Wild-type (WT, n = 16) and t-PA^-/-^ mice (n = 9) were immunized with the MOG_35-55_ peptide to induce EAE. Disease severity was evaluated daily using a neurological severity score scale (see methods). As shown, t-PA^-/-^ mice developed EAE symptoms at a greater rate than WT mice. Data presented as Mean±SEM. *p<0.05; **p<0.01; ***p<0.001. (2-way ANOVA). The data from (A) was also graphed in terms of percentage symptom-free (B) and percent survival (C) post-immunization. For C, percent survival was based on mice with a neurological severity score (NSS) less than 3. As shown, a greater proportion of t-PA^-/-^ mice displayed less symptom-free survival (p<0.001) and significantly reduced survival rates (p<0.01) following EAE compared to wild-type control mice. (Gehan-Breslow-Wilcoxin test).

EAE was induced by immunization with the myelin oligodendrocyte glycoprotein (MOG_35-55_) peptide [34]. Mice received 200 μg of MOG_35-55_ peptide (GL Biochem, Shanghai China) emulsified in Complete Freund’s adjuvant (CFA) supplemented with 1 μg of *Mycobacterium tuberculosis* (H37 Ra; DICO Laboratories. USA) subcutaneously on Day 1. Mice were injected intraperitoneally with 0.35 μg of reconstituted *Bordatella pertussis* toxin (Sigma, Dorset, UK) in 200 μl of phosphate buffered saline (PBS) at both 24 h and 48 h after MOG_35-55_ immunization. Mice were monitored and weighed daily. Clinical disease was assessed and scored based on a neurological severity score (NSS) [35]: 0, normal; 1, limp tail; 2, impaired righting reflex; 3, paresis of hind limbs; 4, complete paralysis of hind limbs; and 5, moribund/dead as previously described [36]. The activity of animals was monitored over 5 minutes. Increments of 0.5 were used to accommodate mice with symptoms between scores. According to our ethics approval, all mice that underwent induction for EAE were not maintained beyond a NSS of 3 (i.e. were culled). This was designated the ethical upper limit of the experiment. Hence, we were unable to score mice with a NSS greater than 3 and mice that attained this level of severity were deemed to have maintained this NSS level for the entire monitoring period. Also, when evaluating “percent survival” following EAE, this was based on mice with an NSS score of less than 3. Mice showing symptoms were given fresh mashed food daily and easier access to water in addition to regular maintenance. Mice were killed either by CO_2_ asphyxiation (first two cohorts of mice, Fig 1) or by terminal urethane injection (all other cohorts) as approved by our animal ethics committee when they reached a NSS of 3 or on day 40, whichever came first. Spinal cords were collected after transcardial perfusion (TCP) with either PBS or with 4% paraformaldehyde. Spinal cord, plasma and brain were removed, prepared and stored at -70°C until used. Control mice were non-injected animals of similar age, sex and relevant genotype.

Note, the use of urethane was specifically approved by our animal ethics committee for terminal euthanasia only. Urethane was administered by intraperintoneal (i.p.) injection at a dosage of 250 mg/ml (~250 μl) and administered in a well-ventilated room designed for animal experimentation. When urethane used, appropriate safety measures were used, including the use of gloves. For additional safety measures and as required by our Institutional Ethics committee, urethane was also kept is a locked cabinet.

### Tissue preparation

Spinal cord and brain samples were homogenized with 1% Triton-X 100 in PBS pH 7.2, adjusting lysate concentration 150mg (wet weight tissue) per ml. Cell debris was removed by centrifugation and total protein concentration was measured using the Pierce BCA Protein Assay (ThermoScientific #23225). Analyses were performed according to the manufacturer’s instructions, and final protein amount was expressed as mg/ml.

### Western blot analysis

Western blot analysis was performed to detect fibrinogen levels in brain and spinal cord extracts of wild-type and EAE mice. Protein samples (50 μg) were subjected to SDS-PAGE electrophoresis under denaturing conditions and transferred to nylon membranes using standard conditions To detect fibrinogen, membranes were hybridized with a polyclonal rabbit anti-human fibrinogen antibody (DAKO, Code A 00801 diluted 1:1000) that cross-reacts with mouse fibrinogen. After washing, membranes were hybridized with HRP tagged secondary goat anti-rabbit IgG antibody. Fibrinogen signals were revealed using enhanced chemiluminescence (SuperSignal West Pico; Thermo Scientific, Rockford, IL. USA).

### SDS-PAGE fibrin zymography

Fibrin zymography was performed using 10% SDS-PAGE gels as previously described [37]. 50 μg of protein (or 1 μl of undiluted plasma) was loaded per well and electrophoresed under non-reducing conditions. After washing the gels in 3 x changes of washing buffer, gels were overlaid onto low-melting agarose matrices containing polymerized plasminogen-free fibrinogen (1%, Enzyme Research Laboratories, USA) and human plasminogen (1 μg/ml final concentration; Enzyme Research Laboratories, USA). Agarose-Gel assemblies were placed in a humidified plastic container and transferred to a 37°C incubator. Lysis zones were visualized at regular intervals over a 4 day period.

### Gelatin zymography

Gelatin zymography and Coomassie blue staining was used to detect MMP-2 and -9 activity in brain and spinal cord extracts (20 μg) as previously described [31].

### Densitometry

X-ray films (western blots) and gelatin zymograms were scanned and saved as 8-bit grayscale files. Quantification of band intensities was carried out using ImageJ densitometry plug-in (National Institutes of Health).

### S-2251 amidolytic assay

The S2251 amidolytic assay was used to determine the rate of t-PA-mediated plasmin generation in brain and spinal cord tissue as previously described [38]. Briefly, 20 μl of each spinal cord lysate was transferred to an ice-cooled 96-well plate. A reaction buffer was prepared containing S-2251 and cyanogen bromine-digested human fibrinogen (CNBr-fibrinogen) at final concentrations of 2 mM and 0.1 mg/ml, respectively. CNBr-fibrinogen acts as a fibrin co-factor and accelerates the reaction. Plasminogen was then added to a final concentration of 0.5 mM and the reactions made up to a final volume of 50 μl per sample with PBS. A multichannel pipette was used to dispense 50 μl of this reaction buffer into each well, and 50 μl mineral oil was then placed on the top of each well to minimize loss through evaporation. Absorbance at *λ* = 405 nm was measured every 2 minutes for 6–8 hours at 37°C using a fluorescence plate reader (BMG Fluostar Optima). For quantitative data analysis of the reaction containing plasminogen, all values beyond substrate depletion were first excluded. Second-order polynomial equations were best fitted to each ‘absorbance at *λ* = 405 nm *vs*. time’ curve using GraphPad Prism Version 6.0 software. The second-order coefficient of each best-fit polynomial equation was taken as half the initial rate of the amidolytic assay.

### RNA isolation and reverse transcriptase-PCR

Mice were killed at NSS 3 or at day 40. Following transcardial perfusion a section of spinal cord 2 cm from the brainstem was removed. Total RNA was extracted using RNAeasy Lipid Tissue mini kit (374804; Qiagen, USA) following the manufacturer’s instructions. The optional DNase digestion steps were included. RNA quantification was analysed using the nanodrop ND-100 spectrophotometer (ThermoFisher Scientific USA). From each sample, 1 μg purified RNA from each sample was used for reverse transcription (RT; Invitrogen, San Diego, CA). cDNA (50 ng) from each spinal cord was then used for qPCR in a final volume of 7 μl. t-PA was detected using gene expression assay Mm00476931_m1 (Life Technologies). Duplicate cDNA templates were processed in a 7900HT Fast Real Time PCR system using the following program: Initial denaturation 5 min at 94°C then 35 amplification cycles of [denaturation (94°C, 30 secs), annealing (60–65°C, 30 sec), and extension (74°C, 45 sec)]. The level of each cDNA was quantified in the exponential phase of PCR product accumulation and normalized to the level of the HPRT housekeeping gene.

### Statistical analysis

The unpaired t-test, one way ANOVA (Neuman-Keuls) was used to assess the significance (p<0.05) of results. Survival analysis of series was performed using Survival curve analyses with Gehan-Breslow-Wilcoxon t-test. This was performed using GraphPad Prism version 6.0 software. Statistical results for clinical observations were presented as two-tailed t-tests.

## Results

### Comparison of EAE severity and rate of onset in wild type and t-PA^-/-^ mice

Wild-type mice and t-PA deficient mice (male) were subjected to MOG_35-55_ immunization at the same time, and the onset, rate and severity of EAE evaluated over a 30 day period. Wild-type mice showed visible signs of EAE by day 9, with the severity of disease varying and the majority reaching NSS 2 by day ~17. In contrast, t-PA^-/-^ mice showed an earlier disease onset and on average manifested a more severe form of clinical symptoms with most mice reaching NSS 3 between days 12–15 (Fig 1A). When evaluating these data in terms of days symptom-free it was also apparent that the entire t-PA^-/-^ cohort had initiated disease by day 10, whereas 50% of the wild-type mice were symptom free up until day 15 (Fig 1B, p<0.001). These data were also presented as “percent-mice with NSS <3” (Fig 1C), further confirming that t-PA^-/-^ mice exhibit enhanced disease progression (p<0.01). The orginal data files are presented in S1 Fig.

These results agree with the report by East et al [21] yet contrasts with the earlier study by Lu et al [20] who reported a delay in symptom onset in t-PA^-/-^ mice. Nonetheless, as this study and the two previous studies all revealed greater disease severity in t-PA^-/-^ mice it is apparent that endogenous t-PA serves a protective role during the development of EAE.

### t-PA activity levels increase in the spinal cord of wild-type mice following EAE

**Fig 2:**
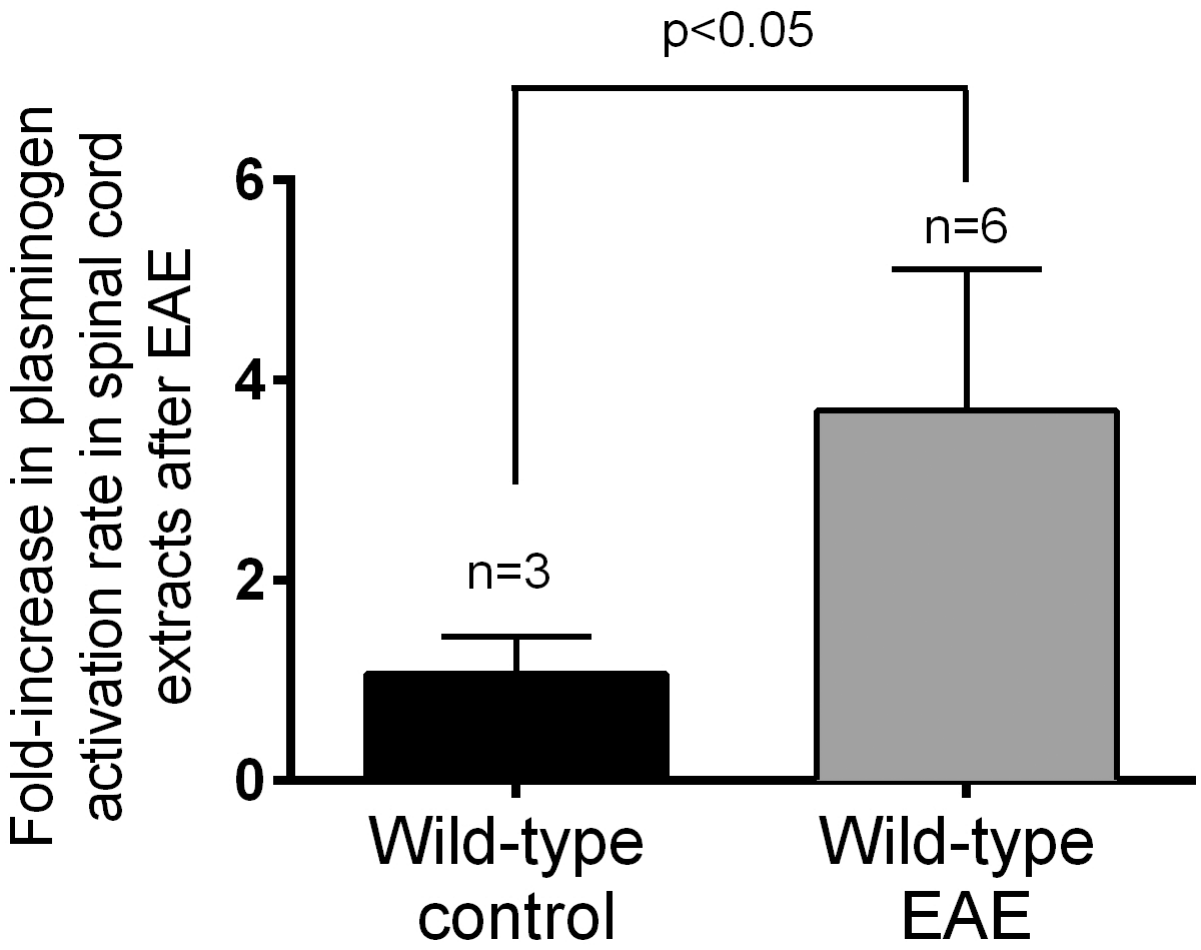
Endogenous levels of t-PA activity in the spinal cord increase following EAE. Changes in t-PA activity were determined by amidolytic assays in spinal cord extracts of wild-type mice at NSS stage 3 after MOG_35-55_ immunization. As shown, EAE causes a significant increase in levels of endogenous t-PA activity, n = 3–6. Data has been normalized to non EAE controls (control) and presented as Mean±SEM.

To determine whether endogenous t-PA activity was altered in mice following EAE onset, amidolytic assays were performed using spinal cord extracts from wild-type mice that attained an NSS of 3. As shown in Fig 2, the rate of plasminogen activation in spinal cord extracts increased ~3.7-fold in wild-type mice subjected to EAE relative to their non-treated controls. Original data files for Fig 2 are presented in S2 Fig. Plasminogen activation was not evaluated in t-PA^-/-^ mice immunised with the MOG_35-55_ peptide as our earlier study [38] showed that unchallenged t-PA^-/-^ mice have no detectable amidolytic activity in the CNS.

**Fig 3:**
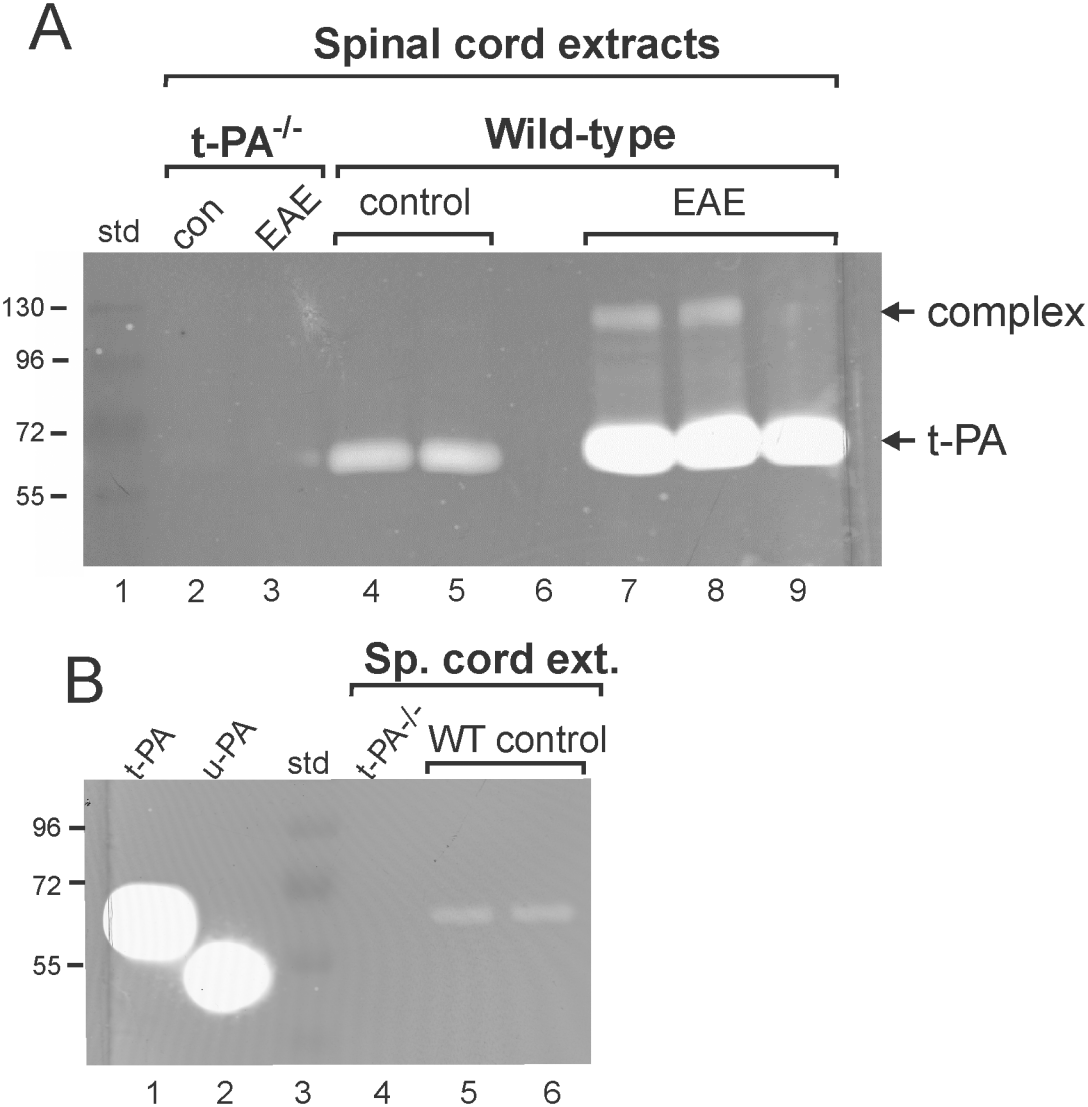
Fibrin zymographic analysis of t-PA activity in spinal cord extracts of mice subjected to EAE. (A): Spinal cord extracts (50 μg protein loaded per lane) from MOG_35-55_ immunized t-PA^-/-^ and wild-type mice at NSS stage 3 of EAE were subjected to SDS-PAGE electrophoresis and fibrin zymography. As shown, wild-type mice demonstrated an increase in t-PA activity following EAE. Also evident is the presence of a slower migrating fibrinolytic moiety present following EAE most likely representing t-PA:PAI-1 complex formation. (B): Fibrin zymogram performed using t-PA and u-PA standards showing that the plasminogen activator activity seen in spinal cord extracts from wild-type (WT) control mice co-migrates with t-PA (note that the samples run in lanes 5 and 6 in (B) were the same samples loaded to lanes 4 and 5 in (A). Extracts from t-PA^-/-^ mice were also used as a control to indicate the absence of both t-PA and u-PA.

To further evaluate changes in t-PA-induced plasminogen activation following EAE induction, spinal cord extracts were subjected to fibrin zymographic analysis. Spinal cord lysates of wild-type mice showed a clear increase in t-PA activity following EAE induction relative to their non-EAE counterparts (Fig 3A). Although this increase was mainly seen for free-t-PA, an increase in a slower migrating fibrinolytic band was also evident (top arrow in Fig 3A). The molecular weight of this band is suggestive of tPA:PAI-1 complex formation and increases in PAI-1 have been reported in the spinal cords of mice subjected to EAE [21]. To confirm that the fibrinolytic activity was indeed due to t-PA, another experiment was performed where the same control samples in Fig 3A were subjected to SDS-PAGE fibrin zymography (Fig 3B) with the inclusion of standards for both t-PA (lane 1) and u-PA (lane 2). It is apparent from the migration profiles that the fibrinolytic activity present in the spinal cord extracts co-migrates with t-PA (Fig 3B). Increases in t-PA activity were also seen in brain homogenates, but the magnitude of change was less (not shown). As expected, no detectable t-PA activity was found in spinal cord samples from t-PA^-/-^ mice (Fig 3A lanes 2 and 3; Fig 3B lane 4).

### MMP-9 levels are increased in both wild-type and t-PA^-/-^ mice following EAE

**Fig 4:**
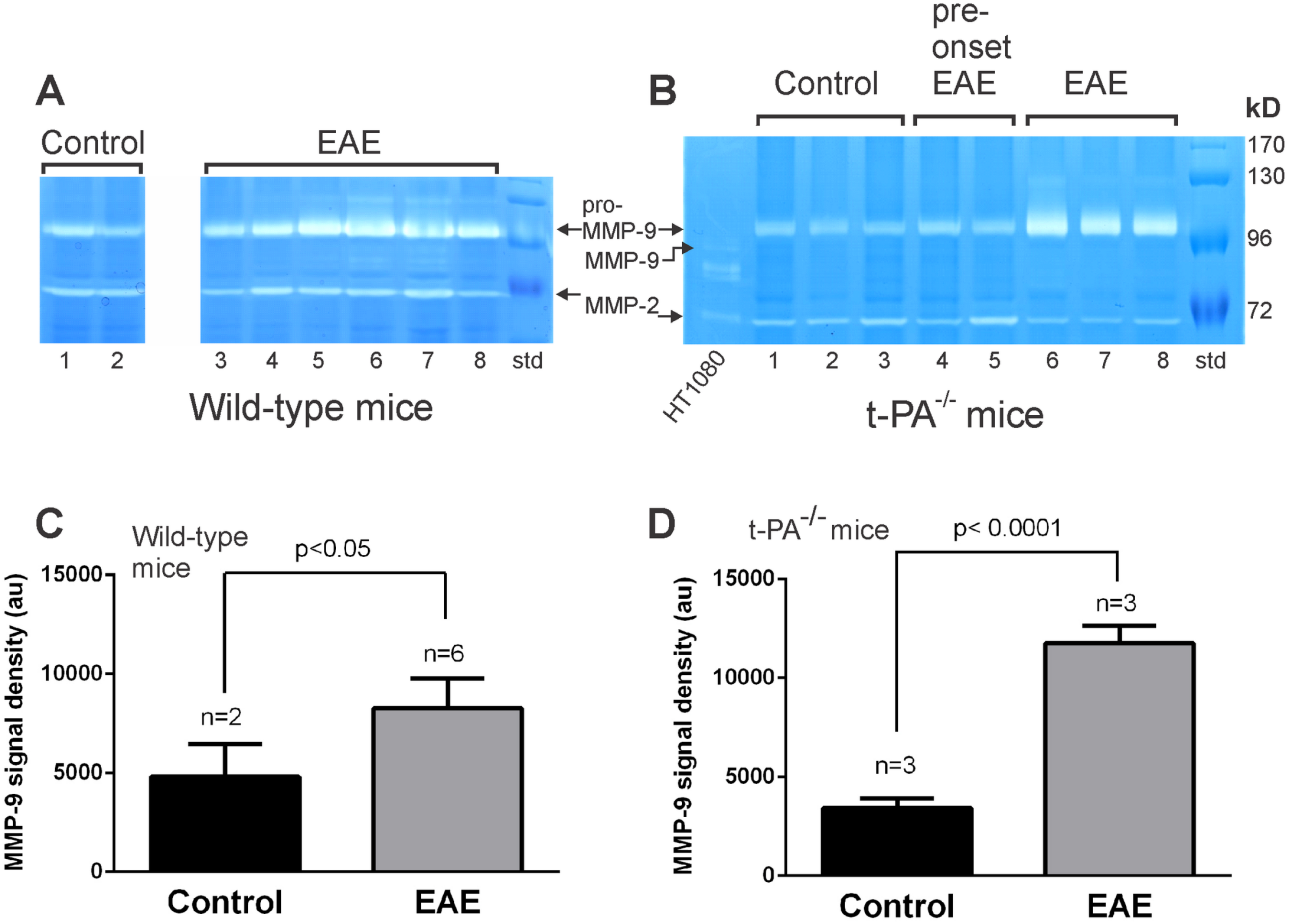
MMP-9 levels are increased in spinal cord extracts from wild-type and t-PA^-/-^ mice following induction of EAE. Spinal cord extracts (20 μg per lane) from MOG_35-55_ immunized t-PA^-/-^ and wild-type mice at NSS stage 3 of EAE were subjected to SDS-PAGE electrophoresis and gelatin zymography. As shown, both wild-type and t-PA^-/-^ mice demonstrated an increase in proMMP-9 but there was no significant change in levels of MMP-2. The increase in proMMP-9 was not seen in t-PA^-/-^ mice at an early stage of EAE (pre-onset, NSS stage 1.5). As a control for active MMP-9 activity, conditioned media from HT1080 fibrosarcoma cells were included that constitutively express pro-MMP-9 (left lane of B). pro-MMP-9 in the HT1080 samples was activated using amino-phenyl mercuric acetate (APMA) to convert pro-MMP-9 into its active form (shown on the gel). The signal intensities (in arbitrary units; “au”) for MMP-9 in wild-type (A) and t-PA^-/-^ mice (B) were quantitated by densitometry and presented in 4C and 4D, respectively.

The matrix metalloproteinase family of proteases have also been associated with the progression of EAE. Moreover, the plasminogen activating system is known to activate a number of MMP family members, including MMP-2 and -9. To determine whether there were substantial changes in MMP expression following EAE onset and if this was altered in mice t-PA^-/-^ mice, gelatin zymographic analyses were performed on spinal cord extracts (20 μg/lane) from both WT and t-PA^-/-^ mice that reached NSS stage 3 after immunization. MMP-2 activity in spinal cord lysates in WT mice were not significantly altered in either wild-type or t-PA^-/-^ mice subjected to EAE and this was confirmed when evaluated by densitometry (Fig 4A: wild-type mice con v EAE: p = 0.405; Fig 4B: t-PA^-/-^ mice con v EAE p = 0.1266). In contrast, levels of MMP-9 were increased in wild-type mice following EAE (~1.7-fold, Fig 4C) and more substantially increased in t-PA^-/-^ mice (~3.2-fold; Fig 4D) following EAE. It remains to be determined if the greater fold increase in MMP-9 in the t-PA^-/-^ mice compared with wild-type mice is a genuine consequence of t-PA deficiency. The original data files for Fig 4C and 4D are presented in S3 Fig.

We also included conditioned medium from HT-1080 fibrosarcoma cells that constitutively express MMP-9. These samples were further subjected to activation by amino-phenyl mercuric acetate (APMA) which is a standard procedure for converting pro-MMPs into their active forms [39, 40]. As shown using the active HT-1080-derived MMP standards (Fig 4B), it is evident that the MMP-9 detected in spinal cord extracts is present in its pro form (proMMP-9), and hence is not active.

### Comparison of EAE severity in mice neuronally over-expressing endogenous t-PA

**Fig 5:**
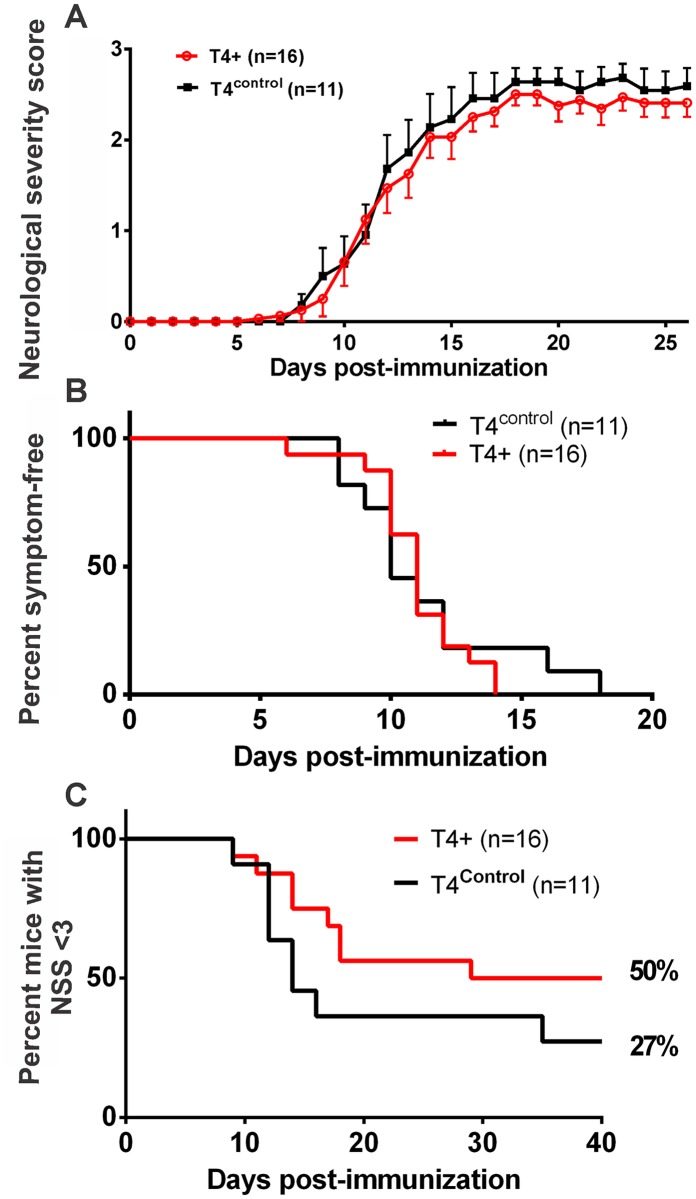
T4+ mice and littermate T4^control^ mice display a similar degree of disease severity following induction of EAE. T4+ transgenic mice overexpressing mouse t-PA in neurons (n = 16) and wild-type littermate control mice (T4^control^, n = 11) were immunized with the MOG_35-55_ peptide and the rate of EAE onset and severity determined. There was no difference in the change in neurological severity over time (A; p>0.05 for all time points), percentage of mice symptom free between the genotypes (B; p>0.05). T4+ mice showed a non-significant increase in survival rate following EAE (C; p >0.05). Note that the data in (C) shows mice that survived with a neurological severity score (NSS) less than 3. Statistical tests used in this figure was Gehan-Breslow-Wilcoxon t-test. This was performed using GraphPad Prism version 6.0 software.

We next assessed whether a constitutive increase in the level of endogenous t-PA in the CNS would protect mice against EAE. To address this, EAE was induced in T4+ transgenic mice and their littermate controls (^T4control^ mice) and the time of disease onset and severity determined. The time-dependent change neurological severity following MOG_35-55_ immunization in both the T4+ and T4^control^ mice was similar with mice of both genotypes showing initial signs of impairment between day 7 and 8 after immunization while both groups attained NSS 2 after 14 to 15 days (Fig 5A). This is also highlighted in Fig 5B where both the T4^control^ and the T4+ mice showed similar symptom-free disease incidence with all mice in both groups developing EAE symptoms between days 14 and 16 (Fig 5B). Interestingly, T4+ mice showed an apparent survival benefit (based on percent mice with NSS less than 3) with 50% of T4+ mice reaching severe acute EAE by day 40, compared to 73% of T4^control^ mice, but this was not statistically significant (Fig 5C). The original data files for Fig 5 are presented in S4 Fig. Hence, overexpression of t-PA in the CNS of mice does not appear to confer any significant protection against either the time of onset or severity of EAE.

### Comparison of t-PA and MMP activity and levels in spinal cord of T4 mice following EAE

**Fig 6:**
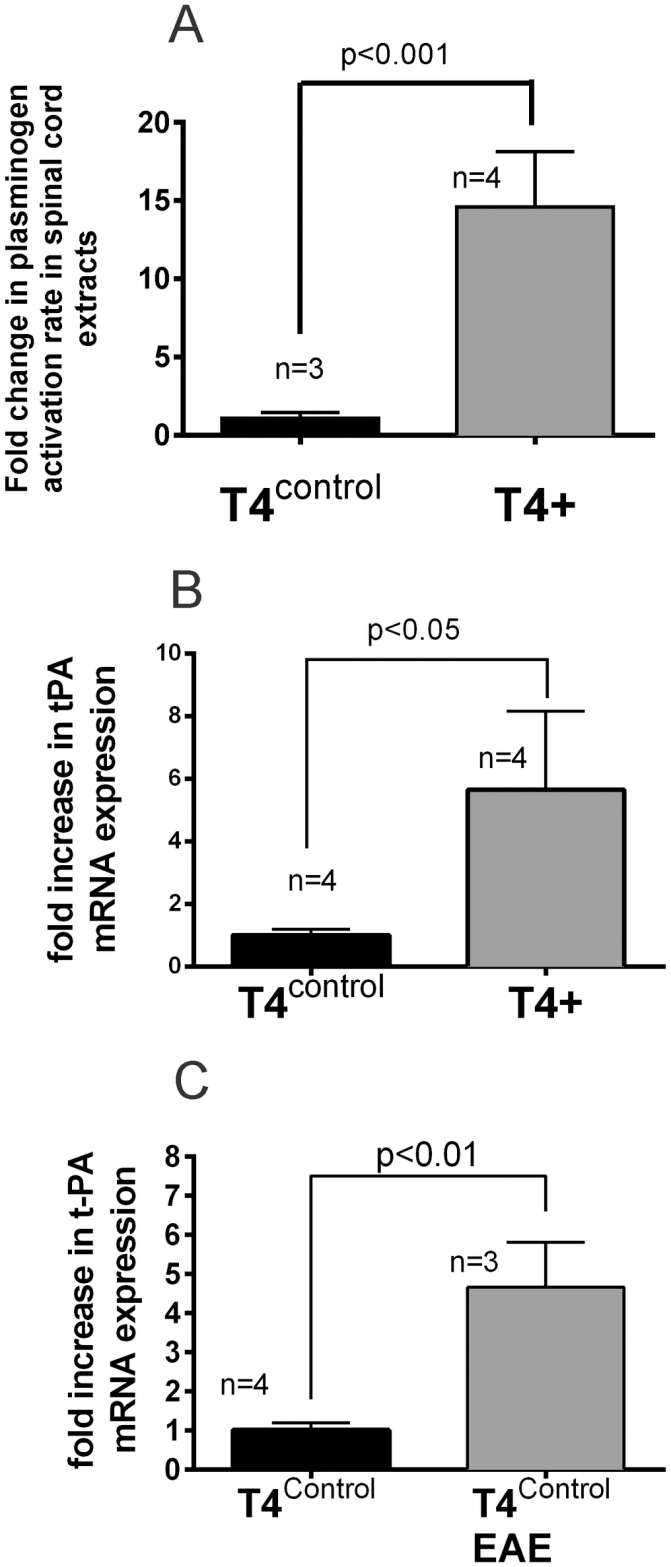
t-PA activity and mRNA levels are increased in spinal cord extracts of T4+ mice. **A**: Spinal cord extracts prepared from naïve T4 ^control^ and T4+ mice were evaluated for t-PA dependent plasminogen activation using an amidolytic assay (see methods). T4+ mice (n = 4) displayed a 14.58 ± 1.78 fold increase in plasminogen activation rates relative to their littermate T4^control^ mice (n = 3). Data presented as Mean±SEM. *p<0.001. As described in the methods section, samples were adjusted to 20 μg/ml and 20 μl used for this determination.**(B)**: A ~4-fold increase in t-PA mRNA is also observed in spinal cord extracts from naïve T4^control^ and T4+ mice (p<0.05). Data presented as Mean±SEM.**(C)**: T4^control^ mice immunized with the MOG_35-55_ peptide also displayed a ~4-fold increase in t-PA mRNA levels (assessed at clinical severity score of 3); p<0.01. Data presented as Mean±SEM.

To confirm that the T4+ mice displayed an increase in endogenous t-PA expression in spinal cord extracts [41] under naïve conditions, t-PA activity and mRNA levels in spinal cord extracts were evaluated using amidolytic assays, and by qPCR, respectively. As shown in Fig 6A, t-PA activity was increased 14.5-fold in the spinal cord of T4+ mice compared to their T4^control^ littermates under basal conditions, consistent with an earlier study showing an increase in fibrinolytic activity in spinal cord sections in naïve T4+ mice by *in situ* fibrin zymography [41]. Changes in t-PA activity in naïve T4+ mice were also reflected at the mRNA level. t-PA mRNA levels in spinal cord extracts, as determined by qPCR, were increased ~4-fold in wild-type mice following EAE (Fig 6B).

We also evaluated changes in t-PA mRNA levels in the spinal cord of naive T4^control^ mice as well as T4^control^ mice subjected to EAE (and attaining an NSS 3) and observed ~4-fold increase in t-PA mRNA (Fig 6C) consistent with the increase in t-PA activity seen in Fig 2. The original data files for Fig 6 are presented in S5 Fig. We also determined changes in t-PA activity and mRNA levels in T4+ mice subjected to EAE, however no additional increases were seen over naïve levels (not shown). This is not surprising as any change due to disease would be overshadowed by the much greater (~14.5-fold) increase in t-PA expression driven by the transgene.

**Fig 7:**
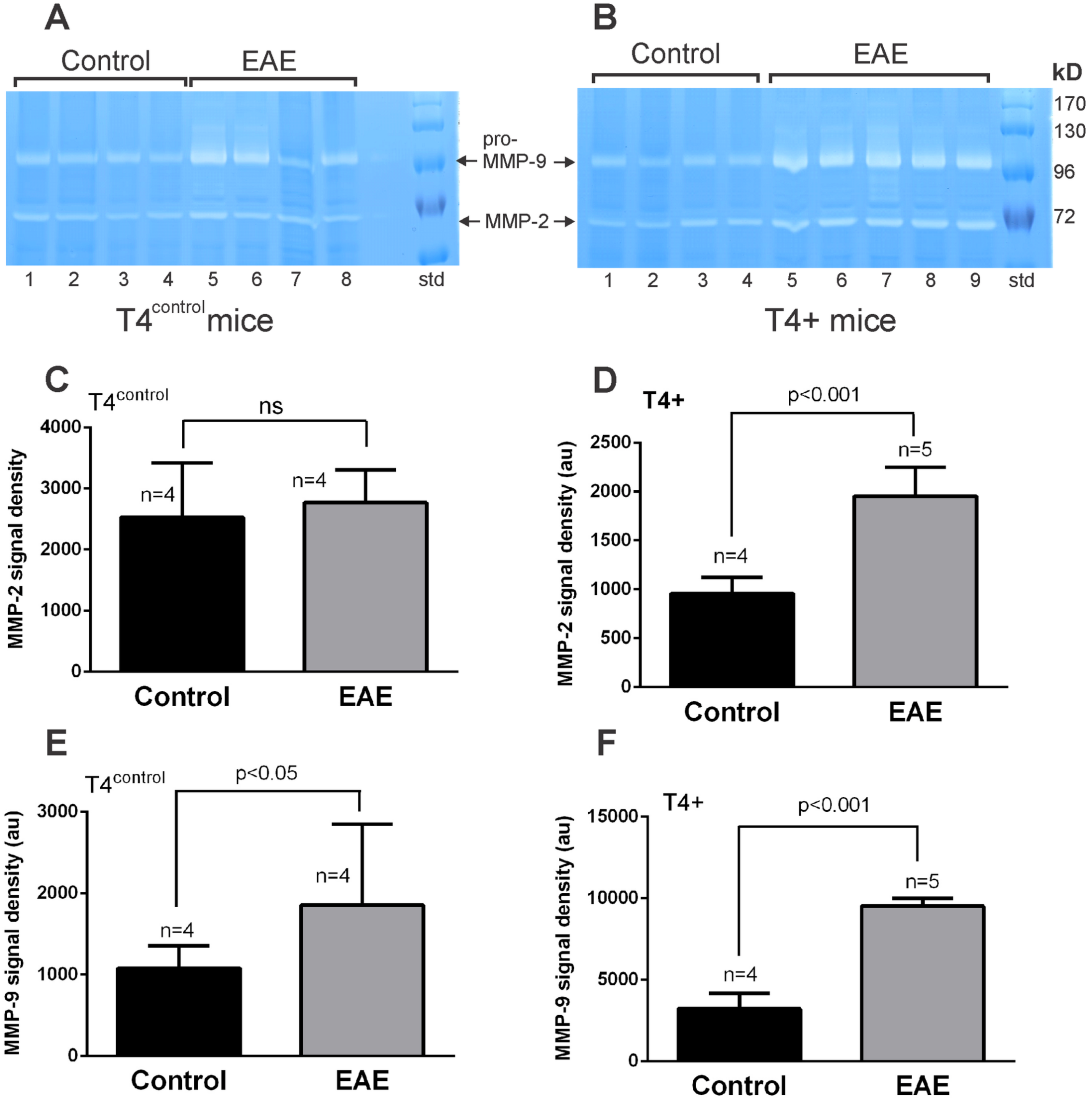
Gelatin zymographic analyses of spinal cord extracts from T4+ mice after EAE. Spinal cord extracts (20 μg protein loaded per lane) from MOG_35-55_ immunized T4^control^ (A) and T4+ mice (B) at NSS stage 3 of EAE were subjected to SDS-PAGE electrophoresis and gelatin zymography. The MMP-2 signals in A were quantified densitometrically. As shown in (C), no significant changes in MMP-2 activity levels are seen in spinal cord extracts from T4^control^ mice following immunization with MOG_35-55_. However, MMP-2 levels are significantly increased in spinal cord extracts from T4+ mice following immunization with MOG_35-55_ as presented densitometrically in D. In contrast, pro-MMP-9 levels in both T4^control^ (A) and T4+ mice (B) were significantly increased in both groups as shown quantitatively in (E) and (F), respectively.

Gelatin zymography was next performed to compare changes in MMP-2 and MMP-9 levels in T4^control^ mice (Fig 7A) and T4+ mice (Fig 7B) following EAE induction. Densitometric analysis of the MMP-2 signals revealed that there was no statistically significant change in MMP-2 levels in T4^control^ mice following EAE at the clinical severity score of 3 (Fig 7C). In contrast, MMP-2 levels were increased (~2.03-fold) in the T4+ mice following EAE (Fig 7D), suggesting a relationship between elevated levels of endogenous t-PA with MMP-2 following EAE. Similar densitometric analyses undertaken for changes in MMP-9 levels indicated significant increases in MMP-9 in both T4^control^ (~1.46-fold, Fig 7E) and T4+ mice (~3-fold, Fig 7F) following EAE. The original data files for Fig 7 are presented in S6 Fig. Hence, both MMP-2 and MMP-9 levels are increased in the spinal cord of T4+ mice following onset of EAE. For MMP-9, this occurs regardless of the absence or over-expression of t-PA whereas the increase in MMP-2 levels in the T4+ mice appears to be related to the overexpression of t-PA.

### Changes in fibrinogen levels in spinal cord extracts following EAE

**Fig 8:**
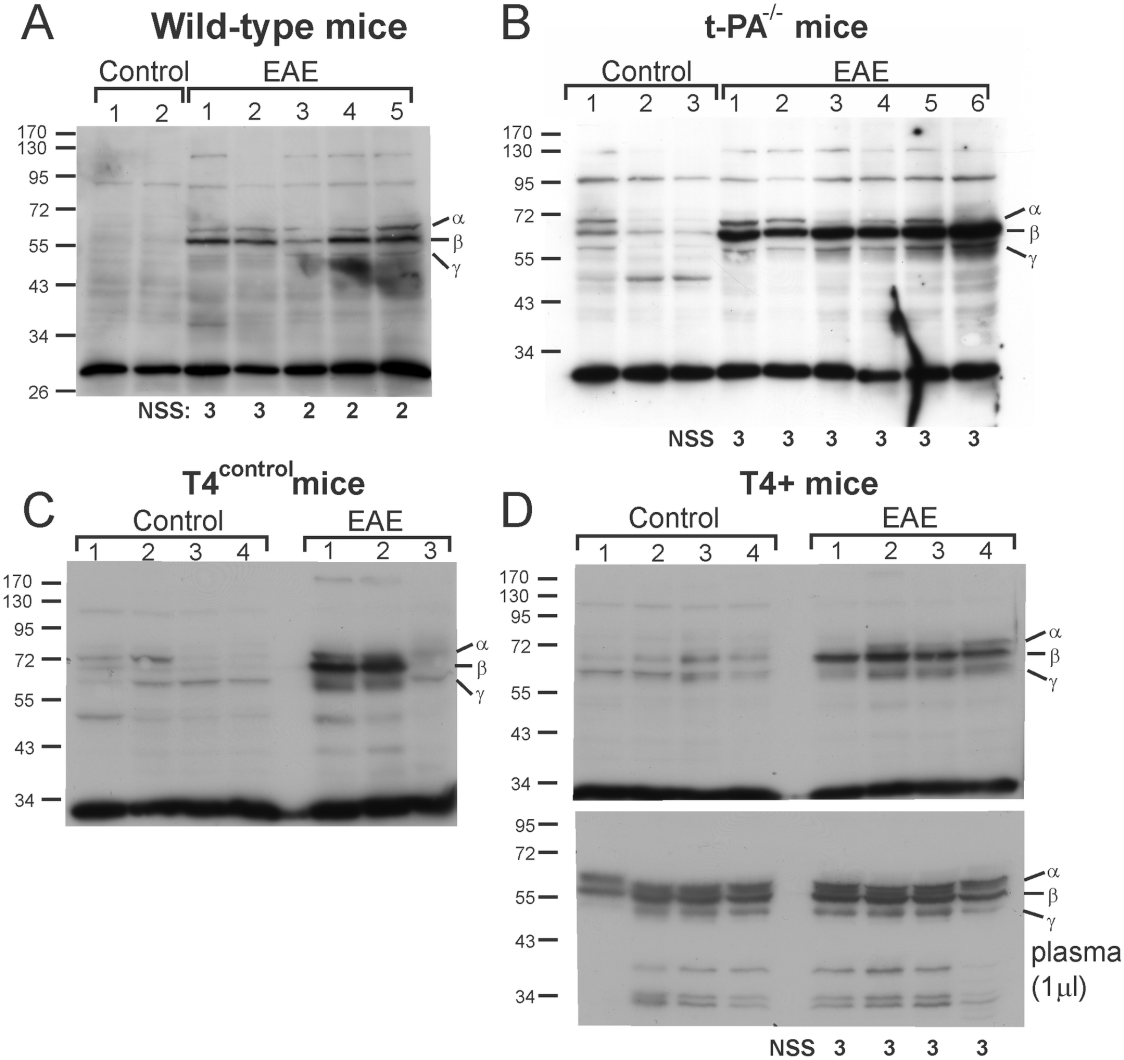
Fibrinogen levels are increased in spinal cord extracts of wild-type, t-PA^-/-^ and T4+ mice following induction of EAE. Spinal cord extracts (50 μg) from wild-type mice (A), t-PA^-/-^ mice (B), T4^control^ mice (C) and T4+ mice (D) were subjected to SDS-PAGE and evaluated for fibrinogen accumulation by western blot analysis under both control (naïve) conditions (CON) and following EAE (NSS 2 or 3 as shown). As shown, all three chains of fibrinogen (α, β, γ) were increased in spinal cords after EAE and this occurred regardless of the absence or neuronal over-expression of t-PA. The lower panel of D is a western blot for fibrinogen in plasma samples (1 μl) from the same T4+ mice as evaluated in the upper panel. As shown, plasma levels of fibrinogen remained unchanged in mice subjected to EAE.

One of the hypotheses underlying this study was that impaired fibrinolysis was promoting EAE severity, most likely by allowing prolonged fibrin formation and accumulation within the lesion. We therefore undertook a series of western blot experiments to evaluate the presence of fibrinogen in spinal cord extracts in all mouse genotypes subjected to EAE. Spinal cord extracts from wild-type, t-PA^-/-^, T4^control^ and T4+ mice following EAE-induction, as well as naïve wild-type mice (i.e. not subjected to MOG_35-55_ peptide immunization), were subjected to 10% SDS-PAGE and western blot analysis by hybridization with a polyclonal antibody against fibrinogen. A marked accumulation of all three chains of fibrinogen: α chain (65 kD); β chain (56 kD) and the γ chain (48 kD) were seen in spinal cord extracts from wild-type mice (Fig 8A), t-PA^-/-^ mice (Fig 8B) and in T4^control^ and T4+ mice after EAE-induction (Fig 8C and 8D, respectively). Densitometric analysis was performed for total fibrinogen levels (all three chains combined) in spinal cord extracts of all genotypes. Fibrinogen levels were significantly increased in t-PA^-/-^ mice at NSS 3 (5.57-fold; p<0.0014) and in T4+ transgenic mice (3.46-fold; p <0.0003). It was also apparent that there was a large increase in fibrinogen levels in wild type mice following EAE, but the fold increase was difficult to ascertain due to the low basal level of fibrinogen in these samples under control conditions.

To rule out the possibility that the increase in fibrinogen was due to a general increase in fibrinogen expression (i.e. due to the acute phase response), fibrinogen levels were evaluated in the plasma of the same T4+ mice as in Fig 8D. As shown in the lower panel of Fig 8D, while there appeared to be a small increase in plasma fibrinogen levels following EAE, densitometric analyses revealed that this increase (1.22-fold) did not reach statistical significance (p = 0.066). Hence, fibrinogen accumulation occurs in the spinal cords of mice subjected to EAE and this occurs regardless of the level of endogenous t-PA.

## Discussion

The primary purpose of this study was to determine whether increased expression of fibrinolytic activity in the CNS served a protective function against EAE. This stemmed from earlier studies reporting significant increases in endogenous t-PA antigen in patients with MS [23, 24, 42]. Similarly, mouse models of MS suggested an association between t-PA expression and MS pathology [43].

t-PA also participates in various aspects of CNS function, including modulation of BBB permeability [25, 38, 44], which allows influx of cells and molecules including fibrinogen into the CNS. Hence, it appears that t-PA could also participate in promoting MS severity by facilitating an increase in BBB permeability while concurrently combating the disease by removing fibrin deposited on demyelinated axons. Evidence for both processes has been reported. Indeed, neuronal overexpression of t-PA (using the T4+ mice) has also been shown to promote extravasation and impair neurological severity in a model of traumatic brain injury [31]. On the other hand, the same T4+ mice were shown to have a protective effect in a model of acute ischaemic stroke [17]. In the context of EAE, it is difficult to predict if overexpression of t-PA would be of benefit (by clearing fibrin) or deleterious (by increasing BBB permeability), or if indeed both processes occur simultaneously or in a temporally distinct manner. Indeed, an increase in plasminogen activation in a rat EAE model and in human MS has been associated with a concomitant increase in BBB permeability [45, 46]. This increase in BBB permeability would also facilitate extravasation of plasma proteins including fibrinogen. In this context, fibrinogen has been shown to deposit as fibrin on damaged axons [45, 47] where it inhibits nerve regeneration [48]. Although t-PA is co-localized with fibrin on axons in MS [25], overall fibrinolytic activity is impaired in MS most likely due to PAI-1 inhibition [30]. As fibrin clearance is correlated with nerve regeneration [29], improving fibrinolysis during MS could provide clinical benefit [47]. However, as mentioned above, this needs to be weighed up against the potential for t-PA to promote BBB opening [11, 31].

Two previous studies have evaluated the onset and severity of EAE in t-PA deficient mice. It was initially reported that t-PA deficiency resulted in a delayed onset but a more severe form of EAE [20]. A later study showed that t-PA deficiency resulted in an earlier onset and also a more severe form of EAE [21]. The reason for these discrepant results with respect to the timing of disease onset is unknown, although different mouse strains may have contributed to this [21]. Nonetheless, both studies concluded that t-PA deficiency cause greater injury severity. Our study fully agrees with the latter study [21] as we showed that t-PA deficiency caused earlier onset and greater injury severity further confirming that t-PA plays a protective role during the host response to EAE. Also as our study used male mice, and the study by East et al used female mice, it is evident that similar penetrance of disease and effect of t-PA on EAE pathogenesis occurs regardless of sex.

The availability of the T4 transgenic mice overexpressing neuronal t-PA provided a novel opportunity to address the effect of neuronal overexpression of t-PA in the EAE model. We hypothesized that overexpression of t-PA may result in a more favourable outcome, possible via improved fibrin clearance from demyelinated axons given that endogenous t-PA plays a protective role in this disorder. However, the T4+ transgenic mice did not display any improved survival rate, suggesting that there is no dose-dependent beneficial effect with excess t-PA. This may also suggest a ‘ceiling effect’ for t-PA, where some t-PA is necessary for protection, increased amounts fail to confer added advantage. In an analogous situation, neuronal expression of t-PA is well known to enhance axon growth [49] yet overexpression of t-PA (using the same T4+ transgenic mice) conferred no protection or improvement of function in following thoracic dorsal hemisection [41]. Although the T4+ mice neuronally overexpress t-PA (~14.5-fold), there remains a possibility that the expression level is too high to be of benefit. Indeed, *in vitro* studies have provided evidence to suggest that low concentrations of t-PA are protective [50, 51] yet toxic at higher levels [52, 53]. However we are unaware of any *in vivo* data showing differential effects of t-PA on neuronal survival. On the other hand, T4+ mice have been reported to be protected in models of ischaemic stroke [17].

We also noted that the degree of severity in T4 control mice (littermates of the T4+ mice) displayed a more severe EAE than was observed with the wild-type Black6 mice (i.e. in Fig 1). Although it was not our purpose to specifically compare the two wild-type cohorts, these different cohorts of wild-type mice were subjected to MOG immunisation months apart and also using a different preparation of the MOG peptide. It remains possible that a more severe disease burden would challenge any potential beneficial effect of neuronally overexpressed t-PA.

Although our findings do not support a protective role for neuronally overexpressed t-PA in the context of EAE, we cannot exclude the possibility that the overexpressed t-PA could act in a beneficial capacity during the remission phase of this disease. Due to ethical restraints, mice that attained a clinical severity score of 3 had to be euthanized. As remission can occur after this clinical score, it remains possible that remission rates may have been more prevalent and/or sustained in the T4+ mice. However, this remains speculative. It was also evident that the T4+ mice presented with a greater increase in survival (50%) compared with the T4^control^ group (27% survival). Although this was not statistically significant, it could be argued that significant differences could be revealed with greater sample sizes. However, in the same cohorts, we observed no significant differences between the T4^control^ and the T4+ mice with respect to the time course of disease onset or in symptom-free survival. Any potential effects of the neuronally overexpressed t-PA on regenerative processes in mice subjected to EAE will require additional experimentation.

Zymographic and amidolytic analyses for t-PA all pointed to an increase in t-PA protein and activity levels in WT mice with established EAE. Nevertheless, some earlier studies reported conflicting results with respect to changes in endogenous t-PA levels following EAE. Two independent reports [21, 25] reported no change in t-PA antigen in spinal cord and brain extracts, respectively, following EAE induction, while conflicting results were reported in another study [20]. While the reasons for these discrepancies are unclear, earlier clinical studies reported that t-PA antigen levels significantly increased (8- to 12-fold) in CSF derived from patients with MS [23, 24].

Fibrin zymographic analyses of spinal cord extracts from mice subjected to EAE not only revealed an increase in levels of free t-PA, but also an increase in the presence of higher molecular weight fibrinolytic complexes. These most likely represent t-PA complexed with its cognate inhibitor, PAI-1 given the molecular weight of this complex), supporting reports of elevated PAI-1 in EAE lesions in mice [21]. It has also been reported that t-PA:PAI-1 complexes are themselves capable of promoting BBB disruption at least in mouse models of traumatic brain injury [31]. Hence it is plausible that such complex formation may also promote BBB opening in MS, but since fibrinogen accumulation in spinal cords was seen to occur in t-PA^-/-^ and T4+ mice argues against a role for t-PA:PAI-1 complex on the BBB in this condition. However, a role for the fibrinolytic system at promoting EAE progression by targeting the BBB in a rat model has been reported [45].

The MMP system is also well-known to play an important role in the pathophysiology of EAE. MMP- 9 levels have been reported to increase in mice following EAE and in the cerebrospinal fluid of MS patients [4, 5, 54] while MMP-9 inhibitors have been shown to be protective in EAE [55]. We observed a clear increase in MMP-9 levels in spinal cord extracts of mice subjected to EAE. While this is consistent with previous reports, our study also demonstrated that MMP-9 levels increased regardless of the absence or overexpression of t-PA. This suggests that the increase in MMP-9 levels is independent of t-PA arguing against a collaborative interaction between the plasminogen activator system and MMP-9 in EAE. Another finding was that the increase in MMP-9 (and MMP-2) in spinal cord lysates seemed to be restricted to their pro- (i.e. inactive) forms, as we did not obtain any clear evidence for an increase in the active form. Whether activation occurs at a later time point remains to be determined. We also note a recent study reporting an increase in MMP-9 activity in spinal cord extracts of mice subjected to EAE. However, MMP-9 activity was only revealed following *ex-vivo* activation with APMA. The prominence of pro- but not active MMP-9 in the spinal cord of mice following EAE may question the role of MMP-9. It is interesting that MMP-9 deficiency has been linked with EAE pathogenesis in young mice but adult mice (as used in our study) appear to lose this effect [5] while administration of MMP-9 inhibitors has been shown to be protective in models of EAE (as reviewed [56]) suggesting that active MMP-9 is indeed important in the manifestations of EAE. It is also plausible that pro-MMP-9 itself could act in a capacity unrelated to its ability to promote proteolysis [57].

Increases in MMP-2 levels in spinal cord extracts of mice subjected to EAE have been reported [58], however in our study, MMP-2 levels in spinal cord extracts were essentially unchanged in wild-type mice and in t-PA^-/-^ mice following EAE (Fig 4A and 4B). Curiously, however, a 2.03-fold increase in MMP-2 levels were seen in T4+ mice following EAE (Fig 7D). This indicates that increases in t-PA coincide with MMP-2 implying co-regulation at least between these two proteases. It would be interesting to determine whether any change in global gelatinolytic activity in mice subjected to EAE correlates with the presence or absence of t-PA.

Our study also demonstrated that EAE causes an influx of fibrinogen presumably as a direct consequence of BBB breakdown. East et al [21] also reported an increase in extravasated fibrinogen (and plasminogen) in spinal cord extracts in mice following EAE, but in that study fibrinogen accumulated to a greater extent (~3-fold) in t-PA^-/-^ mice [21]. This trend was also apparent in our western blot analyses as t-PA^-/-^ mice had a greater fold-increase in fibrinogen compared to T4+ mice. The similar (if not greater) increase in extravasated fibrinogen in spinal cord extracts in t-PA^-/-^ mice and T4+ mice following EAE would argue against a role for t-PA in promoting BBB permeability in this model. However it remains to be determined whether the absence or overexpression of t-PA alters the temporal accumulation of fibrinogen in the spinal cord during EAE progression. A lack of effect for t-PA at promoting BBB permeability during EAE progression contrasts with the results of Patterson et al [45] who demonstrated that BBB permeability in a rat EAE model was reduced by administration of anti-fibrinolytic agents.

In conclusion, this study demonstrates that the absence of t-PA produces a more deleterious outcome in the EAE model of MS, consistent with a previous report [21] and further supports the notion that the endogenous fibrinolytic system contributes to the host response to combat EAE. Surprisingly neuronal overexpression of t-PA, despite expression ~14-fold higher levels of t-PA (Fig 6) confers no any clear advantage to mice against EAE.

## Supporting Information

S1 Fig Prism files related to Fig 1. (PZFX)

S2 Fig Prism files related to Fig 2. (PZFX)

S3 Fig Prism files related to Fig 4. (PZFX)

S4 Fig Prism files related to Fig 5. (PZFX)

S5 Fig Prism files related to Fig 6. (PZFX)

S6 Fig Prism files related to Fig 7. (PZFX)
